# Physiology-Versus Angiography-Guided Complete Coronary Revascularization in STEMI Patients with Multivessel Disease: A Network Meta-Analysis

**DOI:** 10.3390/jcm14020355

**Published:** 2025-01-09

**Authors:** Giovanni Martino, Rossella Quarta, Francesco Greco, Carmen Spaccarotella, Ciro Indolfi, Antonio Curcio, Alberto Polimeni

**Affiliations:** 1 Department of Medical and Surgical Sciences, Magna Graecia University, 88100 Catanzaro, Italy; giovanni.martino004@studenti.unicz.it; 2 Department of Pharmacy, Health and Nutritional Sciences, University of Calabria, 87036 Rende, Italy; rossella.quarta@unical.it (R.Q.); ciro.indolfi@unical.it (C.I.); 3 Division of Interventional Cardiology, Annunziata Hospital, 87100 Cosenza, Italy; francesco.greco@aocs.it; 4 Division of Cardiology, Department of Advanced Biomedical Sciences, Federico II University, 80138 Naples, Italy; carmenannamaria.spaccarotella@unina.it

**Keywords:** percutaneous coronary intervention, physiology-guided PCI, intermediate coronary artery lesions, multivessel coronary disease, STEMI, complete revascularization

## Abstract

**Background**: In patients with ST-segment elevation myocardial infarction (STEMI) and multivessel disease (MVD), complete revascularization (CR) is recommended over culprit-only PCI to reduce adverse cardiovascular outcomes. However, the optimal strategy for CR, whether angiography (Angio)-guided or physiology-guided, remains uncertain. **Methods**: This network meta-analysis included 14 randomized controlled trials (RCTs) with 11,568 patients to compare the efficacy of angio-guided CR, physiology-guided CR, and culprit-only PCI in reducing major adverse cardiovascular events (MACE), all-cause mortality, recurrent myocardial infarction (MI), cardiovascular (CV) death, and unplanned revascularization. The frequentist and Bayesian approaches were applied to assess the effectiveness of each strategy. **Results**: The pairwise meta-analysis showed that angio-guided CR showed superior efficacy, significantly reducing MACE (OR = 0.44; 95% CI: 0.37–0.52), recurrent myocardial infarction, and unplanned revascularization compared to culprit-only PCI. Physiology-guided CR also reduced MACE (OR = 0.64, 95% CI: 0.45–0.91) and unplanned revascularization. The network metanalysis showed that CV death was lower in the physiology-guided CR group (OR 0.56; 95% CI 0.25–1.05), suggesting a protective effect, but the difference did not reach statistical significance. Furthermore, physiology-guided CR was not significantly better than angio-guided CR in most outcomes. **Conclusions**: Angio-guided CR appears to provide the best overall outcomes for patients with STEMI and MVD, outperforming physiology-guided CR in most endpoints. Further large-scale trials are needed to clarify the relative efficacy of angio-guided CR and physiology-guided CR in this patient population.

## 1. Introduction

A substantial proportion of patients (40–60%) with ST-segment elevation myocardial infarction (STEMI) undergoing primary percutaneous coronary intervention (PCI) have obstructive lesions in non-infarct-related arteries (non-IRA) [1,2]. The presence of multi-vessel disease (MVD) has been linked to higher mortality rates in STEMI cases at one year of follow-up, specifically, 3.2%, 4.4%, and 7.8% for single-, double-, and triple-vessel disease, respectively [2]. Complete revascularization (CR) has received a class I recommendation in the ESC guidelines [3] and ACC/AHA guidelines [4]. While there is considerable evidence advocating for CR over culprit-only PCI in patients with MVD-STEMI [5,6], the optimal approach for achieving CR remains a topic of debate [7]. According to current ESC guidelines, PCI on non-infarct-related arteries (non-IRA) is recommended to be guided by angiographic assessment of lesion severity (Class I, level B) [3]. Both angio-guided and fractional flow reserve (FFR)-guided CR have shown improved outcomes compared to culprit-only PCI [8,9]. Nevertheless, head-to-head comparative studies have produced contrasting results about the effectiveness of an FFR-guided approach, making its role in this context unclear [10,11]. This study aims to determine which treatment strategy is most likely to deliver significant clinical benefits while achieving CR and to rank various competing treatments in the context of MVD-STEMI. We employ a network meta-analysis to evaluate angio-guided CR, physiology-guided CR, and culprit-only PCI.

## 2. Materials and Methods

### 2.1. Data Sources and Search Strategy

A computerized search of the MEDLINE, Pubmed, and PMC databases without language restrictions was performed up to May 2024. We used the terms “myocardial infarction”, “non-culprit”, “fractional flow reserve”, “complete revascularization”, “multivessel”, “percutaneous coronary intervention”, “PCI”, and “primary”, both individually and in various combinations to identify randomized controlled trials (RCTs) that evaluated outcomes of culprit-only PCI, physiology-guided complete revascularization (CR), or angio-guided CR in patients with STEMI and multivessel disease up to May 2024. Reference lists of the retrieved studies and the ClinicalTrials.gov registry were also reviewed to identify any relevant studies not captured in the primary search. This meta-analysis followed the guidelines outlined in the PRISMA Extension Statement for Reporting Systematic Reviews that include Network Meta-Analyses [12]. Since this was a study-level meta-analysis, it was exempt from Institutional Review Board oversight.

### 2.2. Selection Criteria

We included RCTs that compared clinical outcomes of culprit-only PCI, physiology-guided CR, or angio-guided CR in patients with acute STEMI and multivessel disease. RCTs that also involved high-risk NSTEMI patients were included in the meta-analysis (STEMI patient threshold was established at 30% as the minimum requirement for study inclusion). For studies with multiple publications, data from the longest follow-up period were used. We excluded non-randomized trials, studies that did not report clinical outcomes, as well as those that exclusively calculated fractional flow reserve (FFR) using the angio-guided method. The quality of randomized trials included was appraised by using Cochrane methods (Supplemental Table S1).

### 2.3. Data Extraction and Outcomes

Two authors (G.M. and R.Q.) independently extracted data from the selected studies. The information gathered included the first author’s name, clinical trial name, publication year, study duration, definitions of multivessel disease (MVD), revascularization strategies, standards for revascularization using physiological guidance, explanations of MACE, observation periods, patient cohorts, baseline study features, and the count of outcomes recorded. Endpoint definitions were based on the criteria established by each individual trial. Any discrepancies among the authors were settled through consensus. The analysis considered only results derived using the intention-to-treat approach. The primary endpoint evaluated was MACE, based on the definitions provided in each study. Secondary outcomes were all-cause death, cardiovascular death, recurrent myocardial infarction, and un-planned revascularization.

### 2.4. Data Analysis

The conventional meta-analyses were conducted using the “meta” package in OpenMetaAnalyst software (version 3.2). For the network meta-analysis, the R package “netmeta” software (Metainsight version 6.0.0) was utilized. A network meta-analysis extends traditional pairwise meta-analysis by allowing comparisons between all treatment pairs within a group targeting the similar condition. Initially, pairwise meta-analyses were conducted to estimate effect sizes in individual treatment comparisons. Following this, a network meta-analysis was implemented through weighted least squares regression to examine the comparative impacts of different therapeutic options. This approach integrates direct and indirect data within a single comprehensive framework. Pairwise network meta-analyses were conducted by computing odds ratios (ORs) and 95% CI using a random-effect (RE) model. The model utilized inverse-variance weighting employing the DerSimonian and Laird methods to address heterogeneity. To analyze the relative effectiveness and ranking of the interventions, we utilized the surface under the cumulative ranking curve (SUCRA) scores. The SUCRA score provides a numerical summary, ranging from 0% to 100%, where a score closer to 100% indicates a higher likelihood that a treatment ranks as the most effective for a given outcome. This approach allows us to compare treatments across a network of interventions, integrating both direct and indirect comparisons. The SUCRA values were computed for each treatment based on their cumulative ranking probabilities, thus enabling a comprehensive assessment of the relative efficacy across all available interventions. The findings were illustrated using forest plots. The variability among the analyses was assessed through τ^2^ and I^2^ statistics. The I^2^ values were interpreted as follows: low (0–25%), moderate (50–75%), or high (>75%) heterogeneity. Sensitivity analyses were conducted by sequentially excluding individual studies to evaluate the stability of the pooled results. Funnel plots were used to assess publication bias. A two-tailed *p*-value of less than 0.05 was considered statistically significant.

## 3. Results

### 3.1. Query Findings

**Figure 1:**
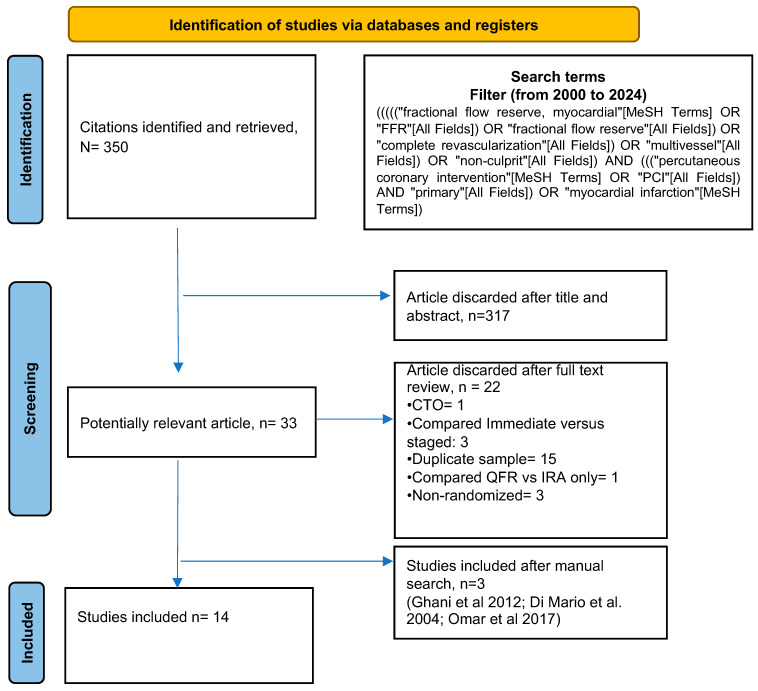
PRISMA (Preferred Reporting Items for Systematic Reviews and Meta-Analyses) flow chart [13,15,20].

**Figure 2:**
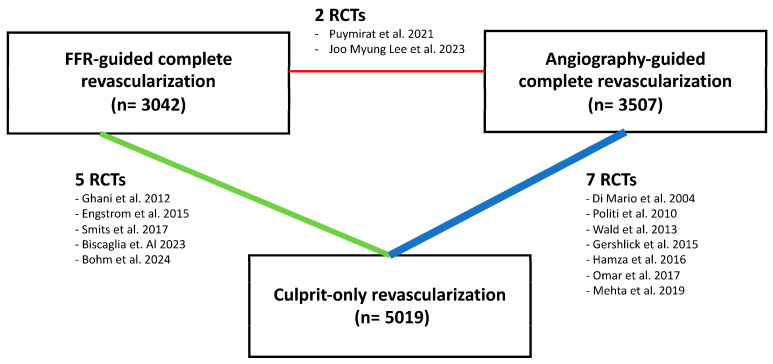
Network plot [8,9,10,11,13,14,15,16,17,18,19,20,21,22].

The initial literature search identified 350 studies that met the predefined eligibility criteria (Figure 1). After a thorough assessment, 14 randomized controlled trials (RCTs) involving 11,568 patients were included in the meta-analysis [8,9,10,11,13,14,15,16,17,18,19,20,21,22]. Of these, 12 studies included 5019 patients receiving culprit-only PCI, 9 studies included 3507 patients receiving angio-guided CR, and 7 studies included 3042 patients receiving physiology-guided CR. Percutaneous coronary intervention was performed if the lesion met the angiographic or physiological threshold and was deemed technically achievable. Figure 2 presents a network plot. Table 1 provides details of the selected 14 RCTs. Definitions of multivessel disease (MVD) varied across these studies, and the follow-up period for the selected trials ranged from six to fifty-eight months. In most studies, physiology was not conducted for stenoses greater than 90%. However, in the FLOWER-MI and FRAME-MI trials, physiology was performed for all stenoses greater than 50% considered eligible for technically achievable PCI; furthermore, in the FIRE-MI, PCI was performed in all lesions with values of iFR was ≤0.89 and QFR ≤ 0.80. Table 2 summarizes the main features of patients involved in the analysis. The patient population was predominantly male (80.52%) with an average age of 63.38 years. Hypertension was observed in 44.96% of the patients, diabetes in 26.13%, and a history of myocardial infarction in 7.27%. In three studies [13,16,20], complete revascularization and coronary physiology measurements were performed during the index procedure. In six studies [9,10,17,18,19,21], these were carried out during the same hospitalization, while five studies [8,11,14,15,22] conducted them at various time points.

### 3.2. Pairwise Meta-Analysis

**Figure 3:**
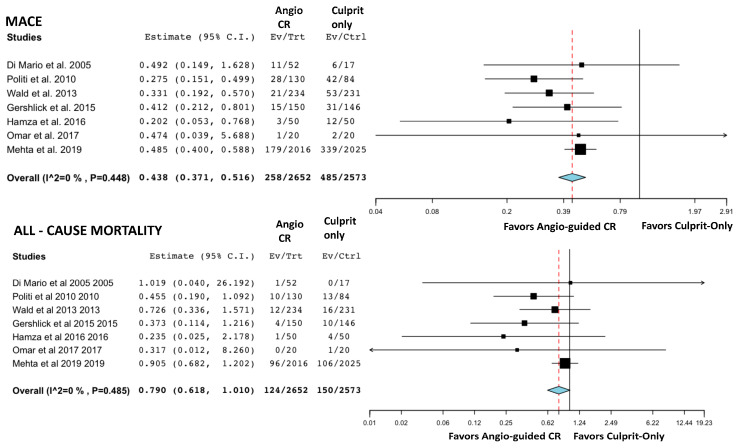

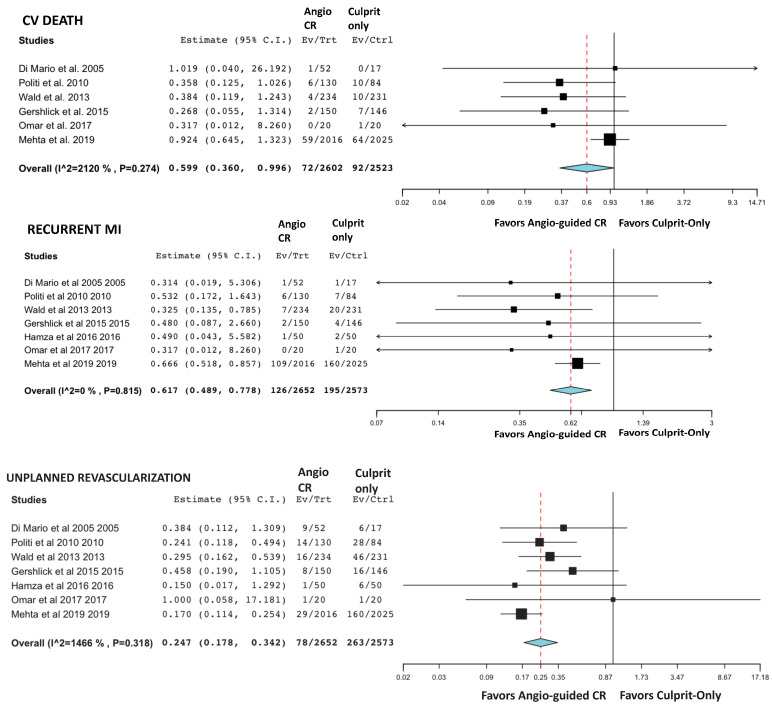
Forest diagrams depicting the outcomes for patients undergoing “Angio CR” or “Culprit-only PCI”. [8,13,14,16,18,19,20]. The red dashed line represents the value of the odds ratio.

**Figure 4:**
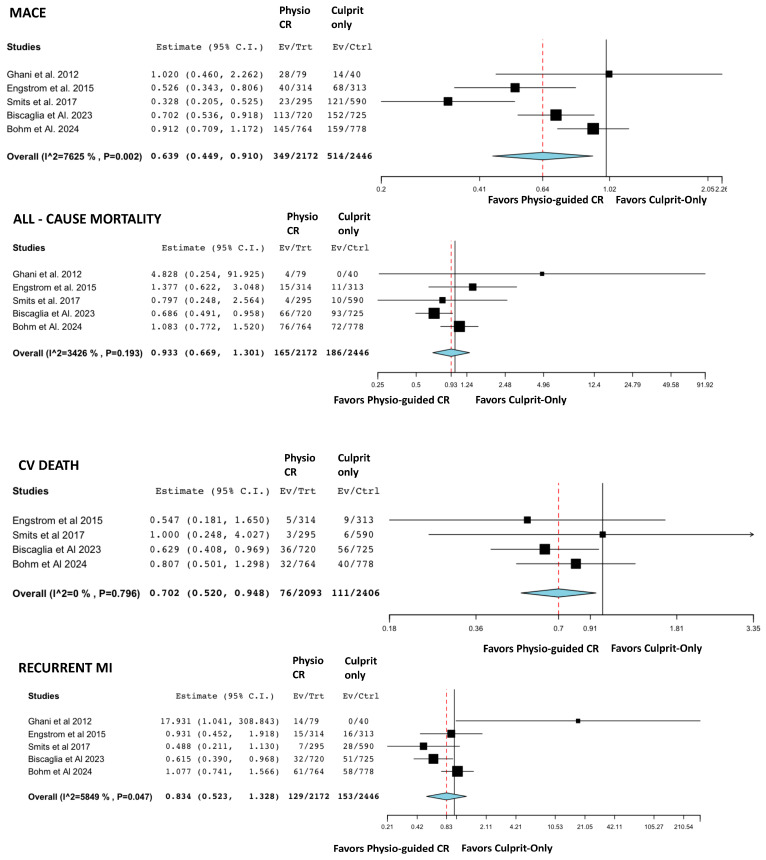

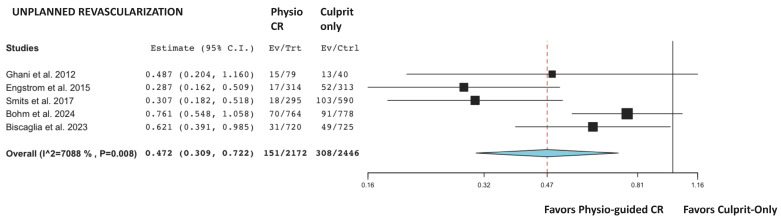
Forest diagrams illustrating outcomes for patients undergoing “Physio-guided CR” or “Culprit-only PCI” [9,15,17,21,22]. The red dashed line represents the value of the odds ratio.

The event counts for each comparison group, along with the findings from the pair-by-pair meta-analyses, are presented in Figure 3 and Figure 4. These figures respectively illustrate the results of the comparisons between angio-guided CR versus culprit-only PCI and physio-guided CR versus culprit-only PCI. The analysis shows that angio-guided CR significantly reduces the rates of MACE (OR = 0.44, 95% CI: 0.37–0.52), recurrent MI (OR = 0.617, 95% CI: 0.49–0.78), and unplanned revascularization (OR = 0.25, 95% CI: 0.18–0.34). Although lower rates of all-cause mortality (OR = 0.79, 95% CI: 0.62–1.01) and CV death (OR = 0.60, 95% CI: 0.36–0.996) were observed, the differences were not statistically significant. Physiology-guided CR demonstrated lower rates of MACE (OR = 0.64, 95% CI: 0.45–0.91), CV death (OR = 0.70, 95% CI: 0.52–0.95), and unplanned revascularization (OR = 0.47, 95% CI: 0.31–0.72) compared to culprit-only PCI. However, no significant differences were found in all-cause mortality or recurrent MI between the two groups. Visual inspection of the funnel plots did not show substantial indication of reporting bias (Supplemental Figure S1).

### 3.3. Network Meta-Analysis

**Figure 5:**
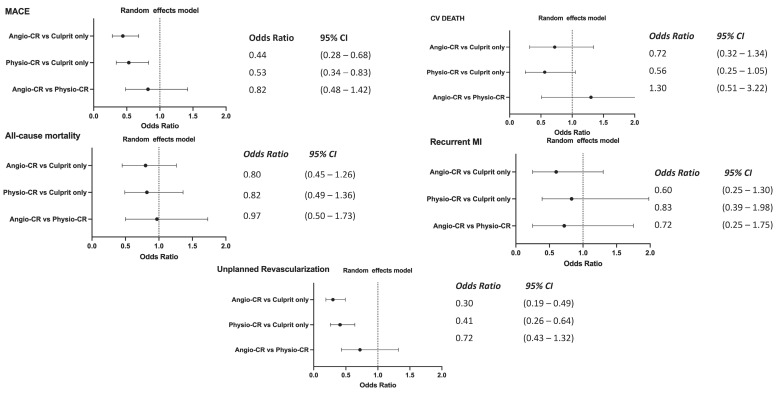
Forest plot showing the relative effects of treatment for each endpoint.

Figure 5 presents the endpoints of the network meta-analysis conducted using the frequentist approach. The findings indicate that both the angio-guided CR and physiology-guided CR groups were linked to significantly reduced rates of MACE and unplanned revascularization relative to the culprit-only PCI group. Although the rates of cardiovascular death were lower in the physiology-guided CR group compared to the culprit-only PCI group (OR 0.56; 95% CI 0.25–1.05), suggesting a protective effect, the difference did not reach statistical significance. Moreover, no significant differences were observed between angio-guided CR and physiology-guided CR across all outcomes.

**Figure 6:**
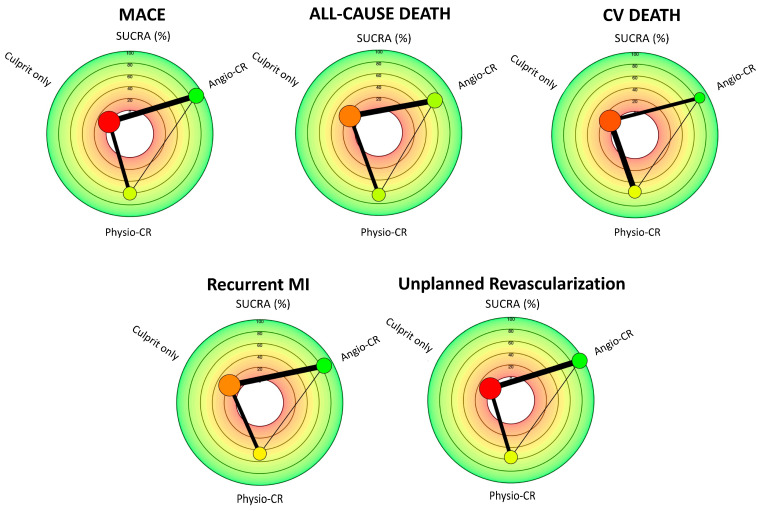
The SUCRA (surface under the cumulative ranking) charts, based on Bayesian network meta-analysis, represent three different treatment strategies: angio-guided complete revascularization (angio-CR), physiology-guided complete revascularization (physio-CR), and culprit only PCI. Each radar plot is associated with a different clinical outcome.

Figure 6 and Table 3 summarize the results of the Bayesian network meta-analysis. According to the SUCRA (surface under the cumulative ranking) charts, angio-guided CR was ranked as the most effective method for reducing MACE (SUCRA score 89%), all-cause mortality (SUCRA score 69%), recurrent myocardial infarction (SUCRA score 85%), and unplanned revascularization (SUCRA score 94%). Physio-guided CR demonstrated competitive performance, with SUCRA scores of 60%, 65%, 46%, and 56%, respectively, particularly excelling in the reduction of cardiovascular death (SUCRA score 86% vs. 56% in the angio-CR group). Culprit-only PCI was ranked as the least effective treatment option for all outcomes.

## 4. Discussion

This network meta-analysis assessed the outcomes of different revascularization strategies in STEMI patients with MVD, utilizing both frequentist and Bayesian approaches. The key findings are as follows: (1) both angio-guided CR and physiology-guided CR led to lower rates of MACE and fewer unplanned revascularizations compared to culprit-only PCI; (2) physiology-guided CR showed a marginally lower incidence of cardiovascular death relative to culprit-only PCI, whereas angio-guided CR did not demonstrate a significant difference in cardiovascular death compared to culprit-only PCI; (3) while no significant differences were identified between angio-guided CR and physiology-guided CR for the analyzed outcomes, angio-guided CR achieved higher SUCRA scores across most outcomes, except for cardiovascular death, where physiology-guided PCI showed a more favorable trend, not statistically significant.

These results challenge the prevalent belief in the superiority of physiology-guided PCI over angio-guided PCI. While prior studies have shown FFR-guided PCI to be more effective than angio-guided PCI in stable patients [23], this result has not been consistently observed in STEMI patients. Although several RCTs have demonstrated the benefits of FFR-guided CR over culprit-only PCI for STEMI patients with MVD, the primary improvements have been driven by reductions in repeat revascularizations rather than hard clinical endpoints [9,15,17]. Furthermore, current evidence comparing angio-guided CR and FFR-guided CR remains limited and often conflicting.

FLOWER-MI and FRAME-AMI were the exclusive RCTs that explored this topic. The first one found no advantage of the FFR-guided strategy in reducing all-cause death and MI in comparison to angio-guided CR, with key limitations including a limited dataset and reduced event rate, which decreased the statistical power. On the contrary, the FRAME-AMI trial demonstrated that FFR-guided CR led to a reduction in the combined outcomes of death, myocardial infarction, and repeat revascularization over a median follow-up period of 3.5 years, compared to angio-guided CR for managing non-culprit lesions in patients with acute MI and MVD. The principal limitation of this study is that it included patients with MVD for both STEMI and NSTEMI cases. Nonetheless, the randomization process was stratified based on the type of myocardial infarction (MI), and no significant interaction was observed between clinical diagnosis and the treatment effect of FFR-guided PCI concerning the primary endpoint. Given the growing prevalence of NSTEMI and its comparable long-term outcomes to STEMI [24], identifying the best approach for achieving complete revascularization in patients with NSTEMI and multivessel disease is also of significant clinical consideration.

The discrepancy between the angiographic and physiological assessment of coronary stenoses is widely recognized. Research has shown that approximately 33% of patients with angiographically significant stenoses exhibited normal FFR values, indicating no physiological abnormality [25]. Evaluating non-culprit stenoses in STEMI patients is particularly challenging, as the angiographic severity of these lesions may be overestimated during an acute event [26]. In contrast, it has been observed that FFR values of bystander lesions in STEMI patients who underwent primary coronary angioplasty were notably greater one month after the STEMI compared to those taken during the acute phase, indicating that stenosis severity may initially be underestimated [27]. Consequently, further research has explored the use of the instant wave-free ratio (iFR) as a reliable approach to guide percutaneous coronary intervention (PCI) in patients with acute coronary syndrome (ACS) and multivessel disease. iFR is calculated during the diastolic phase known as the ‘wave-free period’, which is unaffected by active myocardial contraction or relaxation and during which microvascular resistance reaches its minimum. Thus, iFR assessment can be performed without hyperemic stimulation [28]. Compared to fractional flow reserve (FFR), iFR demonstrates a stronger correlation with coronary flow reserve (CFR), suggesting that iFR may be particularly suitable for assessing stenosis severity in cases where discrepancies exist between FFR and CFR, such as during ACS [28]. Studies have shown that iFR assessment is both feasible and safe, and it is non-inferior to an FFR-guided revascularization strategy with respect to major adverse cardiac events [29,30,31,32]. However, in the acute phase of ACS, iFR may overestimate the severity of non-culprit lesions due to increased resting coronary flow and microvascular dysfunction [27].

Consequently, we performed this analysis to assess the effectiveness of different treatment strategies in STEMI patients with MVD. Unlike traditional pair-wise meta-analyses, this network meta-analysis allowed for both direct and indirect comparisons of interventions across trials by using a common reference. To our knowledge, other network meta-analyses investigating this issue have previously been published [33,34,35]. Our findings are consistent with previous meta-analyses and align with current ESC guidelines, which support angio-guided CR of non-culprit stenoses as the preferred strategy for patients with STEMI. The novelty of our work is to have included in the analysis also the more recent FRAME-AMI, FULL-REVASC and FIRE-MI trials. Unique to our analysis is the observed trend of a reduction in cardiovascular death when the physiology-guided CR is performed. This result may be attributed to the inclusion of a larger number of trials employing the physiology-guided CR strategy within the meta-analysis. This led to a more accurate representation of the patient cohort, providing greater statistical power and a significantly larger sample size compared to previously published meta-analyses.

There are several potential reasons why physiology-guided complete revascularization did not prove superior to angio-guided complete revascularization in our study. The fundamental principle of fractional flow reserve assessment relies significantly on achieving maximal hyperemia, which permits the assumption of a linear relationship between pressure and flow. In STEMI patients, various factors can impair microcirculatory function, thereby hindering the attainment of maximal hyperemia and consequently reducing the accuracy of FFR assessments in non-culprit vessels. These factors include neurohormonal activation, endothelial injury, increased levels of endothelin-1, elevated left ventricular end-diastolic pressure, and myocardial edema [36,37,38]. Together, these elements may lead to temporary impairment of coronary microcirculatory function and diminished responsiveness to adenosine, which can persist for more than a week following the index event [39,40].

## 5. Study Limitations

This study represents an effort to combine existing randomized controlled trials and assess clinical outcomes for three different revascularization approaches in STEMI patients with multivessel disease. Nevertheless, this study is influenced by the limitations inherent in the included trials. Although RCTs exclusively were considered, it was impossible to eliminate entirely potential confounders and selection biases. RCTs that also involved high-risk NSTEMI patients were included in the meta-analysis (the STEMI patient threshold was established at 30% as the minimum requirement for study inclusion). Patients at high risk, including those with severely calcified or tortuous coronary arteries or chronic total occlusions, were typically not included in these studies. As a result, the findings may not be applicable to these groups. Additionally, the definitions of multivessel disease varied across the included trials, leading to heterogeneity in the classification and interpretation of outcomes. Disparities were also observed in the follow-up durations and the descriptions of critical clinical variables, including multivessel disease and physiological cut-off thresholds. Furthermore, the timing of complete revascularization (CR) and coronary physiology assessment varied across studies. The trials spanned a 19-year period, from 2005 to 2024, introducing significant variability in practices, including the use of new generation drug-eluting stents (DES) and potent dual antiplatelet therapy following PCI.

## 6. Conclusions

These results indicate that for patients with STEMI and MVD, angio-guided complete revascularization provides the best overall outcomes for patients undergoing coronary interventions, with physio-guided CR being a viable option for specific outcomes such as cardiovascular death. Culprit-only PCI seems to be the least effective strategy across the board. Further well-powered RCTs are required to definitively establish the relative efficacy of physiology-guided CR compared to angio-guided CR in patients with STEMI. Additionally, there is a need to integrate datasets from real-world studies, such as registries and cohort analyses, to better evaluate factors such as patient adherence, comorbidities, and clinical judgment, which can introduce biases in RCTs. These considerations will help refine the understanding of which CR strategies are most appropriate for specific patient subgroups.

## Figures and Tables

**Table 1:** Main features of the selected RCTs.

Trial, Year	Duration	Criteria of Multivessel Disease	Modality of Revascularization	Criteria for Physio-Guided CR	Description of MACE	Timing of Outcome Evaluation (Months)
Di Mario et al., 2004 [13]	NR	Culprit vessel along with, as minimum, one significant stenosis in a major non-culprit coronary vessel.	Angio-guided CR vs. culprit-only		All-cause death, recurrent myocardial infarction, and repeat revascularization	Twelve
Politi et al., 2010 [14]	2003–07	Stenosis greater than 70% in, at minimum, two epicardial coronary arteries or their major branches.	Angio-guided CR vs. culprit-only		All-cause death, recurrent myocardial infarction, hospitalization for ACS, and revascularization	Thirty
Ghani et al., 2012 [15]	2004–07	Stenosis greater than 50% in, at minimum, two epicardial coronary arteries, or the combination of a side branch and a major coronary vessel supplying distinct myocardial territories.	Physio-guided CR vs. culprit-only	FFR < 0.75 (for stenosis between 50% and 90%) or stenosis greater than 90%.	All-cause death, recurrent myocardial infarction, and urgent revascularization	Thirty-six
Wald et al., 2013 [16]	2008–13	Stenosis greater than 50% in, at minimum, one epicardial coronary artery excluding the culprit vessel.	Angio-guided CR vs. culprit-only		CV mortality, recurrent myocardial infarction, and refractory angina.	Twenty-three
Engstrøm, et al., 2015 [17]	2011–14	Stenosis greater than 50% in, at minimum, one epicardial coronary artery excluding the culprit vessel.	Physio-guided CR vs. culprit-only	FFR ≤ 0.80 (for stenosis between 50% and 90%) or stenosis greater than 90%.	All-cause death, recurrent myocardial infarction, and IDR.	Twenty-seven
Gershlick et al., 2015 [18]	2011–13	Culprit vessel along with, at minimum, one lesion in a major non-culprit coronary artery (stenosis greater than 70% in single view or greater than 50% in two views).	Angio-guided CR vs. culprit-only		All-cause death, recurrent myocardial infarction, hospital admission for heart failure, and repeat revascularization.	Twelve
Hamza et al., 2016 [19]	2013–14	Stenosis greater than 80% in, at minimum, one epicardial coronary artery excluding the culprit vessel.	Angio-guided CR vs. culprit-only		All-cause death, recurrent myocardial infarction, and IDR.	Six
Omar et al., 2017 [20]	2009–11	Stenosis greater than 70% in, at minimum, one epicardial coronary artery excluding the culprit vessel.	Angio-guided CR vs. culprit-only			Six
Smits et al., 2017 [9]	2011–15	Stenosis greater than 50% in, at minimum, one epicardial coronary artery excluding the culprit vessel.	Physio-guided CR vs. culprit-only	FFR ≤ 0.80 (for stenosis between 50% and 69%)	All-cause death, nonfatal myocardial infarction, any revascularization, and cerebrovascular events	Twelve
Mehta et al., 2019 [8]	2013–17	The presence in, at minimum, one angiographically significant non-culprit stenosis, suitable for successful PCI, located in a vessel with minimum diameter of 2.5 mm, and not treated during the index PCI procedure.	Angio-guided CR vs. culprit-only	FFR ≤ 0.80 (for stenosis between 50% and 69%) or stenosis greater than 70%.	CV death, myocardial infarction, or IDR.	Thirty-six
Puymirat et al., 2021 [10]	2016–18	Stenosis greater than 50% in, at minimum, one epicardial coronary artery excluding the culprit vessel.	Physio-guided CR vs. angio-guided CR	FFR ≤ 0.80 (for stenosis greater than 50%)	All-cause mortality, nonfatal MI, and unplanned hospitalization leading to urgent revascularization.	Thirty-six
Joo Myung Lee et al., 2023 [11]	2016–20	Diameter stenosis greater than 50% in, at minimum, one coronary artery excluding the culprit vessel, in a major epicardial artery, or side branch with a minimum diameter of 2.0 mm, judged eligible for PCI.	Physio-guided CR vs. angio-guided CR	FFR ≤ 0.80 (for stenosis greater than 50%)	Death, myocardial infarction, or repeat revascularization	Forty-two
Biscaglia et al., 2023 [21]	2019–21	The presence of at least one lesion in a non-culprit vessel with a visual estimated diameter stenosis between 50% and 99%, in a vessel with a minimum diameter of 2.5 mm.	Physio-guided CR vs. culprit-only	FFR ≤ 0.80, QFR ≤ 0.80, iFR ≤ 0.89	Death, myocardial infarction, stroke, or any repeat revascularization at 1 year.	Twelve
Bohm et al., 2024 [22]	2016–19	Presence of at least one stenosis in a non-culprit artery with a visual estimated diameter stenosis between 50% and 99%, in a vessel with a minimum diameter of 2.5 mm.	Physio-guided CR vs. culprit-only	FFR ≤ 0.80 (FFR was suggested but not mandatory for stenosis between 90% and 99%)	Death from any cause, new myocardial infarction, and unplanned revascularization.	Fifty-eight

Acronyms: NR, not reported; CV, cardiovascular; PCI, percutaneous coronary intervention; CR, complete revascularization; MI, myocardial infarction; MACE, major adverse cardiac events, IDR: ischemia driven revascularization; FFR, fractional flow reserve.

**Table 2:** Patients’ characteristics in the analyzed trials.

Study, Year	STEMI %	Culprit-only	Angio-Guided CR	Physio-Guided CR	Age (Years)	Male (%)	Hypertension (%)	Diabetes (%)	Previous MI (%)	DES Use (%)	Timing of Physiology Measurement/CR
Di Mario et al., 2004 [13]	100%	17	52	N/A	63.9 ± 11.2	87	41	18.8	NR	0	Index
Politi et al., 2010 [14]	100%	84	130	N/A	65.2 ± 12.2	77.6	57.9	19.2	NR	9.8	Index or staged (56.8 ± 12.9 days)
Ghani et al., 2012 [15]	100%	40	N/A	79	62 ± 10	80.2	31.8	5.9	5.8	20.7	Staged (in-hospital or before 3 weeks)
Wald et al., 2013 [16]	100%	231	234	N/A	62 (32–92)	78.1	40.2	17.8	7.5	60.6	Index
Engstrøm, et al., 2015 [17]	100%	313	N/A	314	63 (34–92)	81.1	44.0	11.3	7.0	93.8	Staged (after two days, in-hospital)
Gershlick et al., 2015 [18]	100%	146	150	N/A	64.6 ± 11.2	81.0 (81.1)	36.6	13.6	4.2	93.4	Index or staged (in-hospital)
Hamza et al., 2016 [19]	100%	50	50	N/A	54.3 ± 11.2	84.0	31.0	100	8.0	100	Index or staged (before three days, in-hospital)
Omar et al., 2017 [20]	100%	20	20	N/A	55.2 ± 9.1	82.5	45.0	47.5	20.0	14.3	Index
Smits et al., 2017 [9]	100%	590	N/A	295	61.3 ± 10	77.2	47.2	15.4	7.9	98.8	Index or staged (before three days, in-hospital)
Mehta et al., 2019 [8]	100%	2025	2016	N/A	62.0 ± 10.7	79.8	49.7	19.5	7.5	85.0	Staged 23 (12.5–33.5) days
Puymirat et al., 2021 [10]	100%	N/A	577	586	62.2 ± 11.2	83.1	44.3	16.3	6.5	98.5	Index or staged (2.6 ± 2.4 days, in-hospital)
Joo Myung Lee et al., 2023 [11]	47.2%	N/A	278	284	63.3 ± 11.4	84.3	53.9	32.6	2.5	98.6	During Index-PCI or staged during index hospitalization
Biscaglia et al., 2023 [21]	35.2%	725	N/A	720	80.5 (77–84)	63.4	82.0	32.0	15.2	N/A	Staged 3 days (2–4) in hospital
Bohm et al., 2024 [22]	91%	778	N/A	764	65.4 ± 10.5	76.3	51.2	16.1	8. 1	N/A	During Index-PCI or staged during index hospitalization

Note: Values are mean ± standard deviation, median (interquartile range), or numbers. Acronyms: FFR, fractional flow reserve; DES, drug eluting stent; CR, complete revascularization; MI, myocardial infarction; NR, not reported; N/A, not applicable.

**Table 3:** Comparison of revascularization strategies in STEMI with multivessel disease: results from the network meta-analysis.

Endpoint	Angio-Guided vs. Culprit-Only	Physio-Guided vs. Culprit-Only	Angio-Guided vs. Physio-Guided	Rank Probability (%): Best Approach According SUCRA Score
**MACE**	**OR**: 0.44 (0.28–0.68)	**OR**: 0.53 (0.34–0.83)	**OR**: 0.82 (0.48–1.42)	**1st:** Angio **89%** **2nd**: Physio **60%** **3rd**: Culprit-only **27%**
**All-Cause Death**	**OR**: 0.80 (0.46–1.26)	**OR**: 0.82 (0.49–1.36)	**OR**: 0.97 (0.5–1.73)	**1st:** Angio **69%** **2nd**: Physio **65%** **3rd**: Culprit-only **15%**
**Cardiovascular Death**	**OR**: 0.72 (0.32–1.34)	**OR**: 0.56 (0.25–1.05)	**OR**: 1.3 (0.51–3.22)	**1st**: Physio **86%** **2nd**: Angio **56%** **3rd:** Culprit-only **8%**
**Recurrent MI**	**OR**: 0.6 (0.25–1.3)	**OR**: 0.83 (0.39–1.98)	**OR**: 0.72 (0.25–1.75)	**1st**: Angio **85%** **2nd**: Physio **46%** **3rd**: Culprit-only **18%**
**Unplanned Revascularization**	**OR**: 0.3 (0.19–0.49)	**OR**: 0.42 (0.26–0.64)	**OR**: 0.72 (0.43–1.32)	**1st**: Angio **94%** **2nd**: Physio **56%** **3rd**: Culprit-only **0.3%**

## Data Availability

The data underlying this article are available in the article and in its online Supplementary Materials.

## Supplementary Materials

The following supporting information can be downloaded at: https://www.mdpi.com/article/10.3390/jcm14020355/s1, Table S1: Risk of bias assessment using Cochrane risk of bias tool; Figure S1: Funnel plot for analysis publication bias.
