# Supplementary material for: Physiology-Versus Angiography-Guided Complete Coronary Revascularization in STEMI Patients with Multivessel Disease: A Network Meta-Analysis
Source: J Clin Med. 2025 Jan 9;14(2):355. doi: 10.3390/jcm14020355 (PMC11766365; doi:10.3390/jcm14020355)

MACE Angio-CR vs Culprit-only

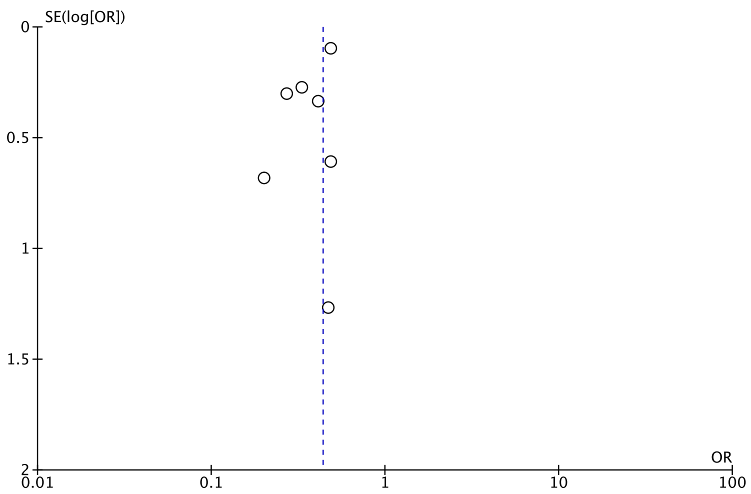

ALL DEATH Angio-CR vs Culprit-only

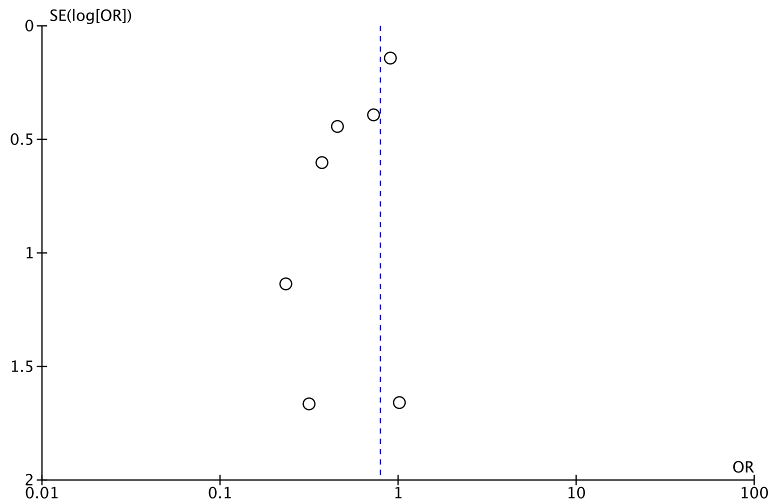

CV DEATH Angio-CR vs Culprit-only

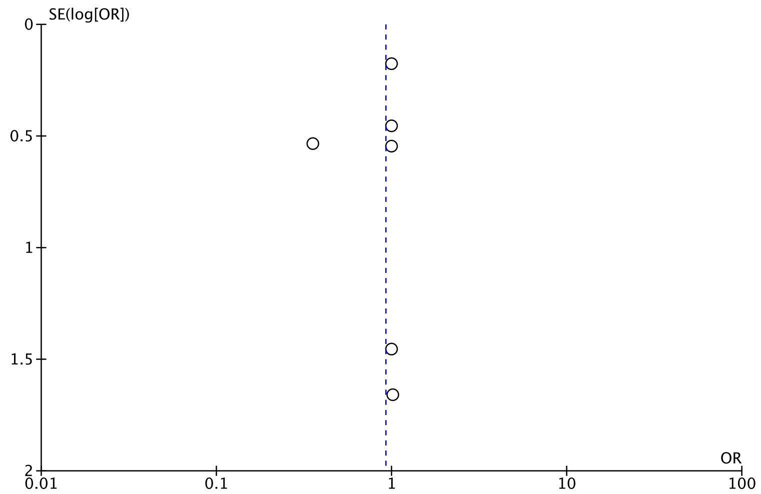

RECURRENT MI Angio-CR vs Culprit-only

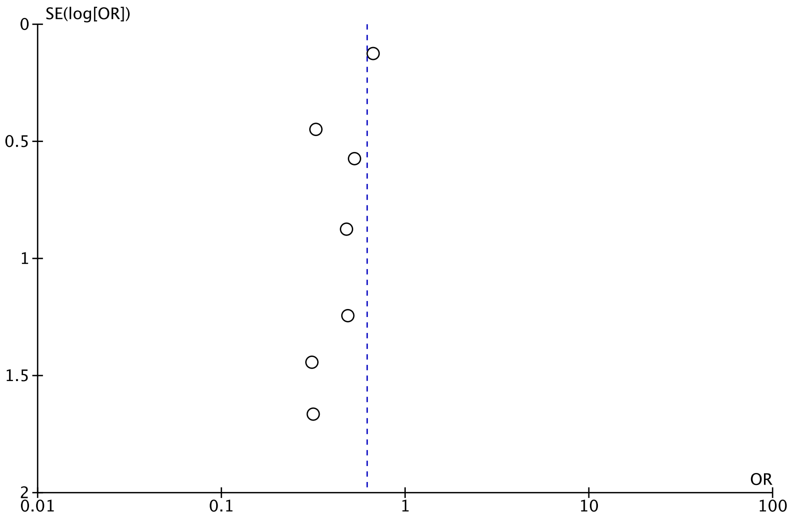

UNPLANNED REVASC Angio-CR vs Culprit-only

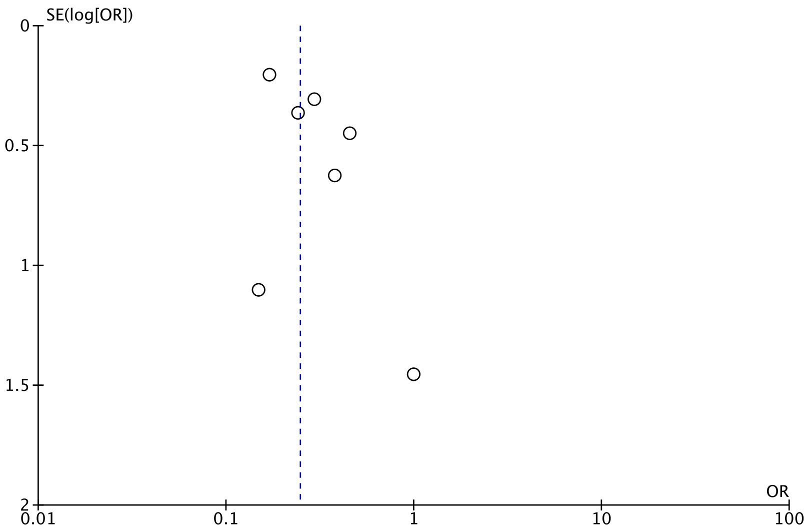

MACE Physio-CR vs Culprit-only

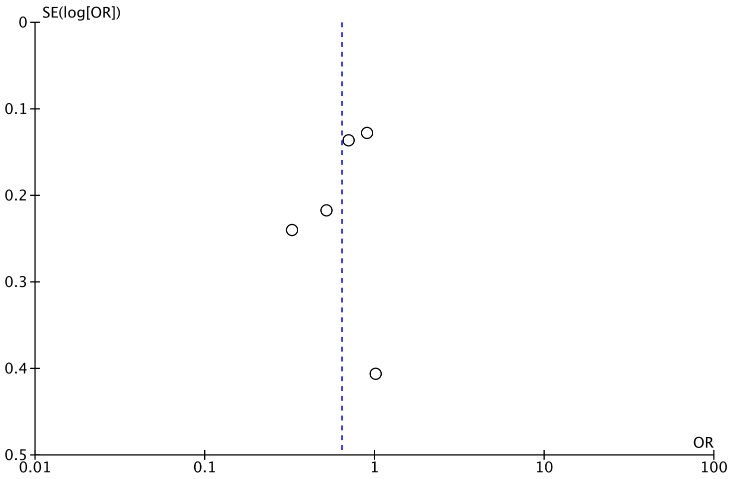

ALL DEATH Physio-CR vs Culprit-only

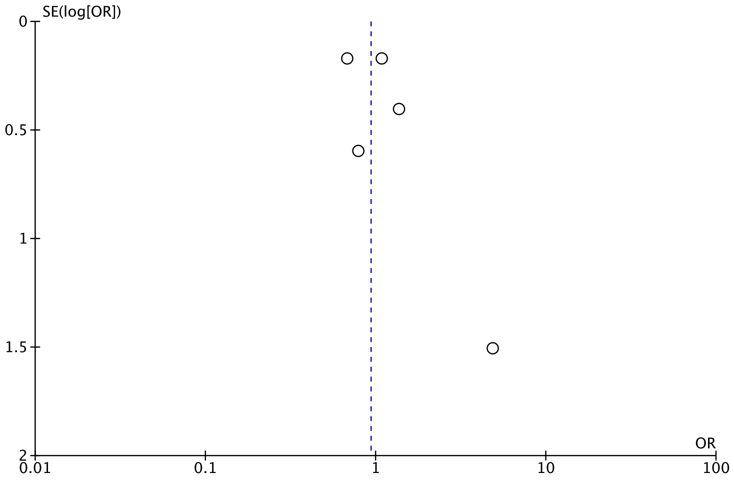

CV DEATH Physio-CR vs Culprit-only

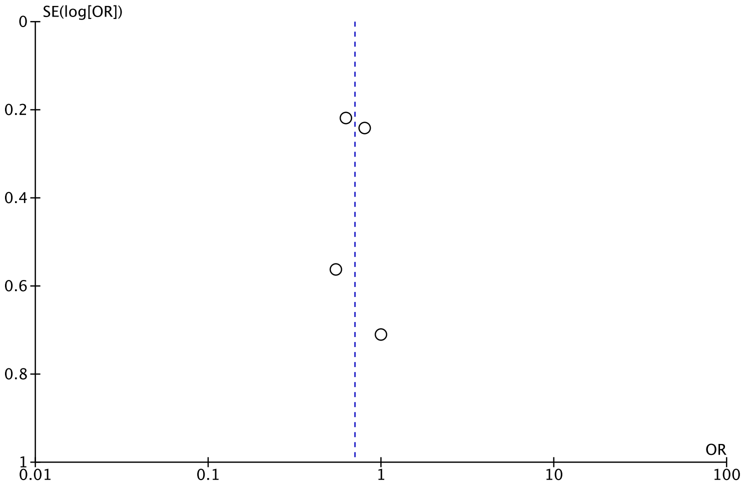

RECURRENT MI Physio-CR vs Culprit-only

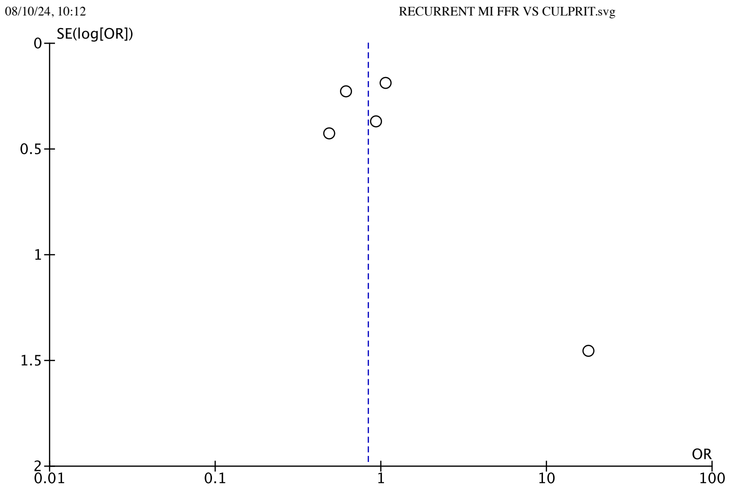

UNPLANNED REVASC Physio-CR vs Culprit-only

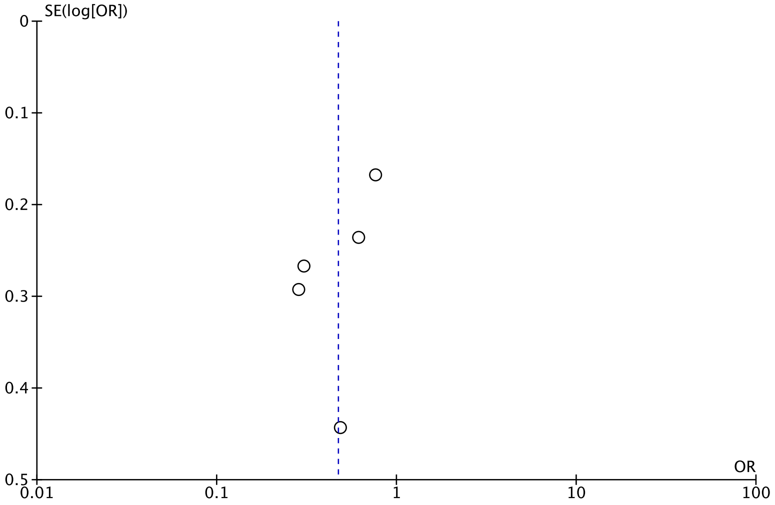

Supplement: Supplementary file 1 [file jcm-14-00355-s001.zip › Figure S1 Funnel plot for analysis publication bias.pdf]
