# Supplementary material for: Physiology-Versus Angiography-Guided Complete Coronary Revascularization in STEMI Patients with Multivessel Disease: A Network Meta-Analysis
Source: J Clin Med. 2025 Jan 9;14(2):355. doi: 10.3390/jcm14020355 (PMC11766365; doi:10.3390/jcm14020355)
Supplement: Supplementary file 1 [file jcm-14-00355-s001.zip › Table S1 supplementary_Cochraine Risk Bias.docx]

| **Study** | **Randomization Bias** | **Deviations from Intended Interventions** | **Missing Outcome Data** | **Measurement of Outcomes** | **Selection of Reported Results** | **Other Bias** |
| --- | --- | --- | --- | --- | --- | --- |
| **Biscaglia et al. 2023** | Low Risk | Low Risk | Low Risk | Low Risk | Low Risk | Low Risk |
| **Bohm et al. 2024** | Low Risk | Low Risk | Low Risk | Low Risk | Low Risk | Low Risk |
| **Engstrom et al. 2015** | Low Risk | Low Risk | Low Risk | Low Risk | Low Risk | Low Risk |
| **Gershlick et al. 2015** | Low Risk | Low Risk | Low Risk | Low Risk | Low Risk | Low Risk |
| **Ghani et al. 2012** | Low Risk | Low Risk | Low Risk | High Risk | Low Risk | Some Concerns |
| **Hamza et al. 2016** | Some Concerns | Low Risk | Low Risk | Some Concerns | Some Concerns | Some Concerns |
| **Help-AMI 2004** | Some Concerns | Some Concerns | Low Risk | Some Concerns | High Risk | Some Concerns |
| **Lee et al. 2023** | Low Risk | Low Risk | Low Risk | Low Risk | Low Risk | Low Risk |
| **Mehta et al. 2019** | Low Risk | Low Risk | Low Risk | Low Risk | Low Risk | Low Risk |
| **Omar et al. 2017** | Some Concerns | Some Concerns | Low Risk | Some Concerns | Low Risk | Low Risk |
| **Politi et al. 2010** | Low Risk | Low Risk | Low Risk | Some Concerns | High Risk | Some Concerns |
| **Puymirat et al. 2021** | Low Risk | Low Risk | Low Risk | Low Risk | Low Risk | Low Risk |
| **Smits et al. 2017** | Low Risk | Low Risk | Low Risk | Low Risk | Low Risk | Low Risk |
| **Wald et al. 2013** | Low Risk | Low Risk | Low Risk | Low Risk | Low Risk | High Risk |

**Table S1**. Risk of bias assessment using Cochrane risk of bias tool.
